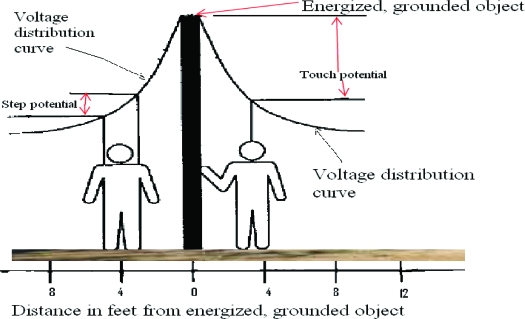# High-Voltage Electrical Shock Without Injury

**Published:** 2011-07-19

**Authors:** Raymond M. Fish

**Affiliations:** Bioacoustics Research Laboratory, Department of Electrical and Computer Engineering, Department of Surgery, College of Medicine, University of Illinois at Urbana-Champaign, Urbana, IL

## DESCRIPTION

A 39-year-old male suffered a minute long shock from a 7200-V power line with no sequelae other than mild quadriceps muscle strain. He had parked a truck next to a highway and was standing on asphalt. He reached into the truck and manipulated a plastic-topped hydraulic lift lever. Suddenly, he could not move or feel. He soon felt a shock and was thrown back. Physical examination was normal except for muscle tenderness.

## QUESTIONS

1. **What body system is most sensitive to electricity?**
2. **A person has a hand-to-foot current path with no electrical contact to the head. Name 3 mechanisms of injury that commonly lead to severe cognitive deficits with such electrical contacts.**
3. **The effects of electricity on the body are mostly related to which of the following: voltage, current, resistance, or impedance?**
4. **A worker picks up a downed power line with an insulated tool rated for the voltage on the line, but is electrocuted. Name 3 reasons why this might happen.**
5. **Contact with overhead power lines causes approximately what percentage of all electrical fatalities of US workers?**

## DISCUSSION

Statistics show that 2287 US workers died and 32 807 workers sustained days away from work because of electrical shock or electrical burn injuries between 1992 and 1998. In those cases, contact with overhead power lines caused 41% of electrical fatalities (Cawley and Homce, 2003).

 High voltage is usually defined as more than 600- or 1000-V 60-Hz alternating current. Many lethal electrocutions have occurred when a person standing on the ground puts his hand on a vehicle that has an elevated part or attachment that is in contact with an overhead power line. Such a hand-to-foot current path includes the heart and spinal cord, but not the head. Brain injury in such cases may result from hypoxia due to respiratory and/or cardiac arrest, carbon monoxide inhalation from fires, falls, and other secondary mechanisms of injury. Most gloves, tools, work boots, and rubber mats will not provide protection from high voltage. Tools and other items that are specially made and rated for high voltages should be tested periodically for tiny defects in insulation, as well as for moisture and other contamination on the surface of the items that could conduct electrical current. Contact with overhead power lines has happened with a variety of vehicles and other devices. There is even a case of a circus employee using an elephant to raise a metal tent pole. The elephant and its trainer were killed when an overhead power line was contacted (Suruda, 1988).

 For electrical current to flow through our patient, it had to go through the plastic knob on the lever, the patient's shoes, rubber shoe inserts, and socks. The high resistance (impedance) of those materials limited the amount of current that would flow. The effects of electricity on the body are related to the amount of current that flows through the various body parts. The amount of current that flows is directly proportional to the voltage and inversely proportional to the total resistance (or impedance) in the current path. In this case, the current was enough to cause loss of muscle control, which takes about 20 mA. Cardiac arrest would be expected around 100 mA, although this varies with the duration of current flow. Thus, the current flow in this case was at most approximately 100 mA or one-tenth of an ampere. As shown in the figure, if there is a downed power line, it is possible to receive a severe electrical shock by simply walking on the ground. When an energized conductor contacts the ground directly or through a conductor, it is referred to as a ground fault. *The decrease of voltage with distance* from the earth contact point of an energized object is called the *ground potential gradient*. Voltage drops associated with this dissipation of voltage are called ground potentials. As shown in the following figure, electric current flows from one foot, is carried through the body to the other foot, and back to ground. With touch potentials, the current goes from one foot, through the body, and to the hand that is touching a grounded structure. The figure and a much more detailed explanation of electrical contacts are in the *ePlasty* article by Fish and Geddes (2009).

## Figures and Tables

**Figure:**
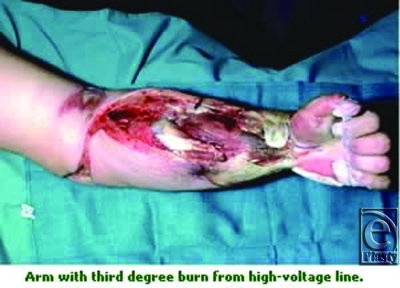


**Figure:**